# An Ecological Mobile Momentary Intervention to Support Dynamic Goal Pursuit: Feasibility and Acceptability Study

**DOI:** 10.2196/49857

**Published:** 2024-03-20

**Authors:** Ciarán O'Driscoll, Aneesha Singh, Iya Chichua, Joachim Clodic, Anjali Desai, Dara Nikolova, Alex Jie Yap, Irene Zhou, Stephen Pilling

**Affiliations:** 1 CORE Data Lab, Centre for Outcomes Research and Effectiveness University College London London United Kingdom; 2 UCL Interaction Centre University College London London United Kingdom

**Keywords:** goal pursuit, ecological momentary intervention, ecological momentary assessment, mood, dynamics, network analysis, MCII, COM-B, support, pilot study, training, feasibility, acceptability, self-monitoring, implementation, psychological, effectiveness

## Abstract

**Background:**

Individuals can experience difficulties pursuing their goals amid multiple competing priorities in their environment. Effective goal dynamics require flexible and generalizable pursuit skills. Supporting successful goal pursuit requires a perpetually adapting intervention responsive to internal states.

**Objective:**

The purpose of this study was to (1) develop a flexible intervention that can adapt to an individual’s changing short to medium-term goals and be applied to their daily life and (2) examine the feasibility and acceptability of the just-in-time adaptive intervention for goal pursuit.

**Methods:**

This study involved 3 iterations to test and systematically enhance all aspects of the intervention. During the pilot phase, 73 participants engaged in an ecological momentary assessment (EMA) over 1 month. After week 1, they attended an intervention training session and received just-in-time intervention prompts during the following 3 weeks. The training employed the Capability, Opportunity, Motivation, and Behavior (COM-B) framework for goal setting, along with mental contrasting with implementation intentions (MCII). Subsequent prompts, triggered by variability in goal pursuit, guided the participants to engage in MCII in relation to their current goal. We evaluated feasibility and acceptability, efficacy, and individual change processes by combining intensive (single-case experimental design) and extensive methods.

**Results:**

The results suggest that the digital intervention was feasible and acceptable to participants. Compliance with the intervention was high (n=63, 86%). The participants endorsed high acceptability ratings relating to both the study procedures and the intervention. All participants (N=73, 100%) demonstrated significant improvements in goal pursuit with an average difference of 0.495 units in the outcome (*P*<.001). The results of the dynamic network modeling suggest that self-monitoring behavior (EMA) and implementing the MCII strategy may aid in goal reprioritization, where goal pursuit itself is a driver of further goal pursuit.

**Conclusions:**

This pilot study demonstrated the feasibility and acceptability of a just-in-time adaptive intervention among a nonclinical adult sample. This intervention used self-monitoring of behavior, the COM-B framework, and MCII strategies to improve dynamic goal pursuit. It was delivered via an Ecological Momentary Intervention (EMI) procedure. Future research should consider the utility of this approach as an additional intervention element within psychological interventions to improve goal pursuit. Sustaining goal pursuit throughout interventions is central to their effectiveness and warrants further evaluation.

## Introduction

### Background

We have all experienced difficulties in developing, pursuing, and achieving our goals. This can be influenced by our internal state (eg, mood and motivation), environmental factors (eg, competing goals or demands), and resources (eg, skills and opportunity). We need the ability to prioritize goal pursuit in a dynamic environment, determining how much effort to allocate and deciding when to shift our attention to other goals [1]. Managing this is a dynamic, within-person process that varies over time based on how much progress one has made toward their goals. These goals or tasks related to goals vary in how demanding they are, and our ability to pursue those goals will be influenced by capacity. Pursuing goals requires effort, both physical and mental, not just due to the difficulty of the task [2] but also for maintaining a mental representation of the goal [3]. Moreover, we need to make decisions around the allocation of resources to the task (eg, breaking down a task or abandoning it), affecting successful pursuit. Effort can be considered synonymous with motivation when measured objectively [4], with motivation influenced by expectancy or certainty and value attributed to the outcome. People exert more effort if the outcome is perceived to be more likely, important, and rewarding [5,6]. In addition, mood, particularly anhedonia, has been associated with behavioral reward processing deficits [4]. The application of theory to intervention suggests a need for a “perpetually adapting” intervention [7].

Successful goal pursuit involves numerous steps: option generation, cost-benefit decision leading to option selection, initiation, and pursuit [8]. Failure at any point can reduce the likelihood of pursuit, and there is a need to anticipate obstacles [9]. By considering obstacles, the individual is better able to anticipate and plan for challenges that may arise as they work toward their goal [10]. The individual must also be able to employ metacognitive strategies such as planning, self-monitoring, and flexibility to overcome challenges, and they may benefit from prompts and support to facilitate these strategies [11].

People’s intentions do not always translate into action: medium-to-large changes in intentions only lead to small-to-medium changes in behavior [12]. Most interventions focus on altering specific behaviors within specific contexts, and the results are not conclusive. Personalized feedback, goal setting, and self-monitoring appear promising, but they are not consistently effective across behaviors and contexts [13]. It is also unclear whether these skills generalize to other behaviors and contexts. Simple strategies or microinterventions can provide easy access and low-effort solutions to increase or maintain engagement in behavior change [14,15]. These strategies may be simple but difficult to sustain without practice.

Ecological Momentary Intervention (EMI) involves providing feedback or intervention to participants in real time based on the data collected from Ecological Momentary Assessment (EMA). The deployment of interventions via mobile devices (eg, smartphones) provides the opportunity to deliver intervention on scale as either a standalone or adjunct intervention. EMA alone can act as a form of self-monitoring, facilitating an awareness of thoughts, emotions, and behavior. From a clinical perspective, the information provided via EMA can support ecological valid assessment and screening, experiential learning, and within the context of an intervention, shed light on the mechanisms of change [16]. Interventions can be personalized, based on momentary assessments, and delivered in anticipation of a change in the target behavior. In the context of adaptive ecological momentary interventions, push/pull strategies involve delivering interventions either proactively by the system (push) or in response to a user’s request (pull), while just-in-time interventions are provided at opportune moments when they are likely to be most effective, based on a real-time assessment of the individual’s context and state [17]. This method can benefit the generalization of skill acquisition, where the just-in-time intervention prompts the individual to allocate increased resources toward skill acquisition.

We propose an intervention that aims to bolster skill acquisition (ie, effective goal pursuit) through a combination of evidence-based strategies and EMI implementation. These strategies include frequent self-monitoring, shown to improve goal attainment [13]; mental contrasting with implementation intentions (MCII) [18], shown to produce a moderate effect on health behaviors [19]; and the Capability, Opportunity, Motivation, and Behavior (COM-B) framework [20] for goal setting. In addition, the intensive measurement of relevant goal-pursuit processes can be used to model the dynamics in daily life [21]. To develop an intervention for implementation within the clinical sample, we optimized the design following a research model for developing digital health interventions through iterations [22].

### Study Aim

This study aimed to develop, evaluate, and implement a just-in-time adaptive intervention to improve goal pursuit. The intervention sought to provide training for a goal pursuit strategy that could easily be incorporated into participants’ daily lives in terms of time and effort. The goal was for this intervention to serve as an adjunct to behavioral interventions (whether (psychological or health-related) in future research.

**Figure 1:**
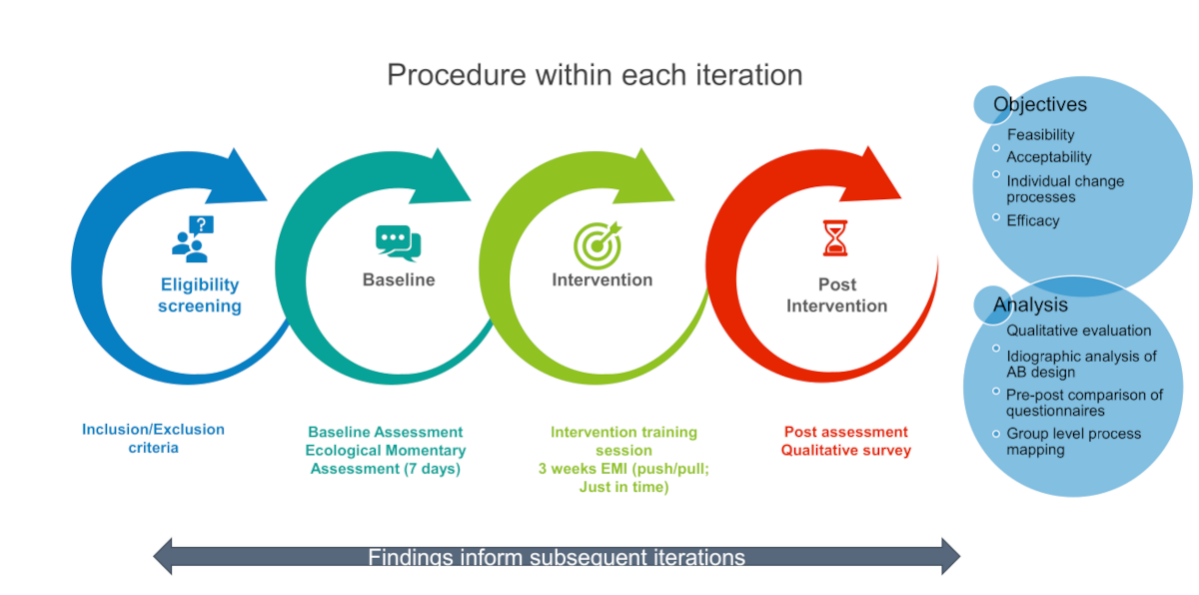
Procedure for optimizing and piloting the ecological momentary intervention, outlining the steps undertaken within each iteration.

The aim was addressed by (1) developing a personalized approach to implementing a goal pursuit intervention in daily life; (2) identifying barriers and facilitators and monitoring the implementation process of the intervention through several iterations; and (3) piloting the intervention to evaluate its feasibility and acceptability, efficacy, and individual change processes by combining intensive single-case experimental design (SCED) and extensive methods. Figure 1 outlines the procedure for optimizing and piloting the ecological momentary intervention via 3 iterations.

## Methods

### Study Design

This study involved 3 iterations to test and gradually improve all features of the intervention to ultimately inform a randomized control trial evaluation of the intervention. Two iterations focused on optimization, while the third iteration was an uncontrolled pilot study. After each iteration, feedback was used to improve the design and intervention to increase the feasibility and acceptability. Questionnaires and strategy training were delivered via Qualtrics and EMA/EMI using the m-path platform [23].

### Ethical Considerations

Ethical approval was granted by the University College London (UCL) Research Ethics Department (21883/001). All participants were provided with details of the study, confirmed eligibility, and provided informed written consent. The study data were deidentified. All participants (including those who chose to withdraw partway through) were entered into a prize draw to win a £50 (US $64) voucher. They received £20 (US $25) and a personalized report outlining their results if they reached 70% completion of the EMA at the end of the study.

### Recruitment

**Figure 2:**
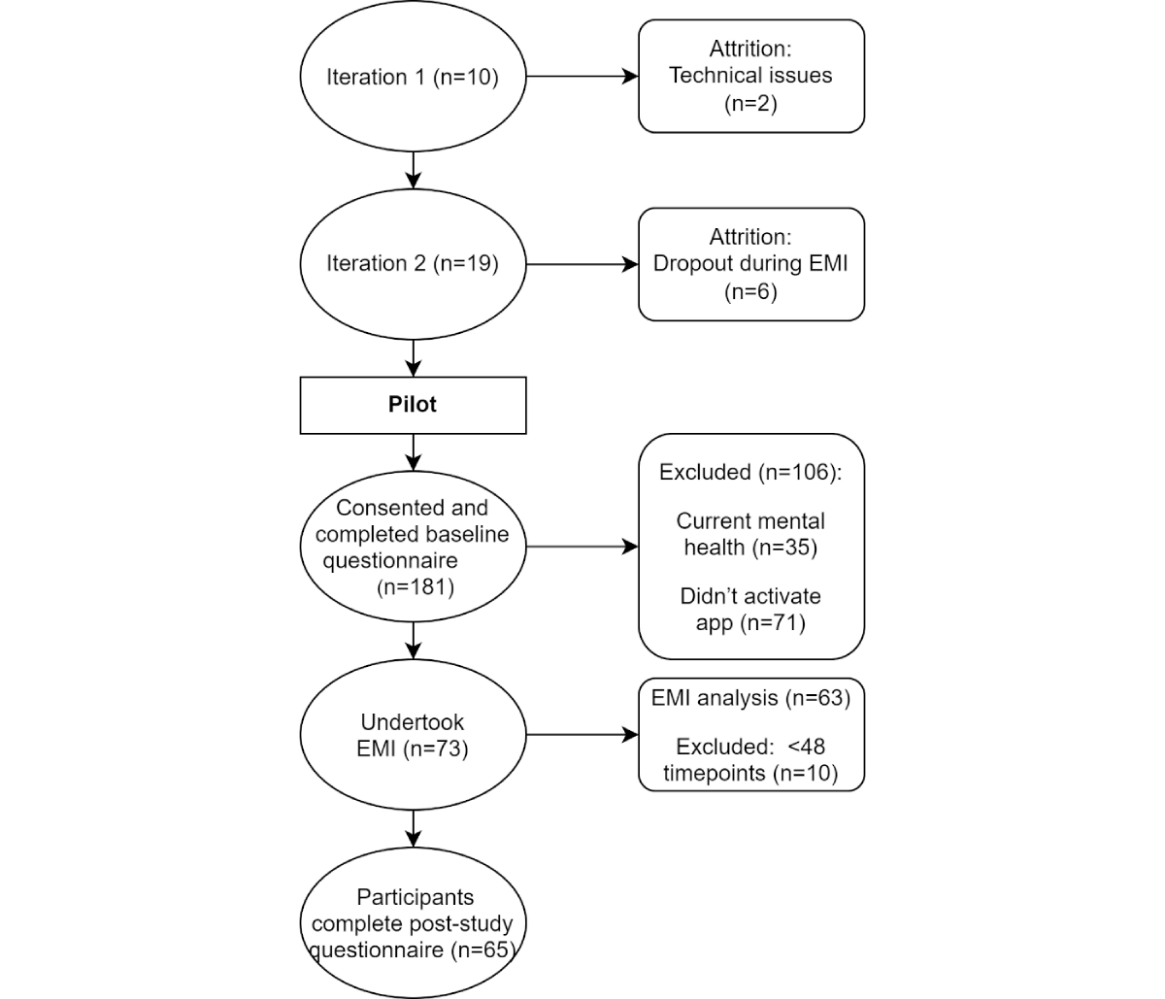
Flowchart outlining the design and participant flow through the development and pilot study. EMI: Ecological Momentary Intervention.

The participants were recruited through a convenience sampling approach from various social media platforms. Individuals were eligible if they (1) were over the age of 18 years; (2) able to read and understand English; (3) were based in the United Kingdom; and (4) had a personal smartphone with Android or iOS operating software. Because we were seeking to pilot in a “healthy” sample, our exclusion criteria were (1) current mental health difficulties, including anyone scoring above 15 on the 9-item Patient Health Questionnaire (PHQ-9) or >1 on the suicidal ideation item and 12 on the Generalized Anxiety Disorder 7 (GAD-7); and (2) anyone undergoing psychotherapy at the time the study was conducted. Figure 2 shows the study flowchart.

EMA studies require commitment from participants over a long period (up to 6 times a day for 28 days); therefore, dropout rates can be high. Appropriate incentives can encourage compliance. Therefore, we created a 2-fold incentive as outlined in the previous section. Participants were also incentivized through app functions such as earning badges and data visualization.

### Developing the Intervention

Developing the intervention relied on adapting existing evidence-based strategies for goal pursuit. We aimed to develop an intervention that was adaptable to each participant’s goals and generalizable enough to accommodate shifts and changes in goal focus during their daily lives. A self-guided training session was developed and supplemented with abbreviated prompts that could be delivered throughout the intervention period (Multimedia Appendix 1).

### Feasibility Assessment

Within each iteration, we collected information relating to the experience of participating in the study, specifically undertaking EMA and the intervention. This included Likert scale questions assessing the ease, helpfulness, and intrusiveness of the EMA; perceived effectiveness and utility of undertaking the intervention; and helpfulness of the strategy training guide. Participants could also provide qualitative feedback on their goal progress and experience. Feasibility was assessed through data relating flow of participant recruitment and retention throughout the intervention. Acceptability was assessed post intervention. This feedback took the form of a questionnaire assessing how feasible and acceptable the EMA schedule was for them; whether it was easy, helpful, or enjoyable; or whether it was intrusive and impeded optimal goal pursuit.

### Assessment of Outcomes

#### Primary Outcome

Goal pursuit was used as the primary outcome to test the preliminary efficacy of our intervention, goal. This was measured using EMA, and participants were also asked about goal attainment post intervention.

#### Secondary Measures

To test whether the intervention influenced goal pursuit–related processes, we assessed participants’ pre-post changes on 5 goal pursuit–related measures, which are outlined in Textbox 1.

**The goal pursuit–related measures we used to assess participants’ pre-post changes.**

1. Action orientation: The Action Control Scale (ACS-90) [24] is divided into 3 subscales: Hesitation (8 items), the ability to initiate a task; Preoccupation (8 items), the ability to actively work on the task; and Volatility (6 items), the ability to stay action-oriented until completion.
2. Defeatist Performance Beliefs: The 15-item Defeatist Performance Beliefs (DPB) scale [25] measures overgeneralized negative thoughts in goal-striving.
3. Prospective imagery: The Prospective Imagery Test (PIT) [26] measures the ability to vividly imagine positive and negative future-orientated scenarios.
4. Intertemporal choice: The 27-item Money Choice Questionnaire (MCQ) [27] measures preferences between small immediate rewards and large delayed rewards. A general delay discounting parameter (“k”) is estimated, wherein greater k values represent steeper delay discounting.
5. Difficulties in Emotion Regulation: The 18-item Difficulties in Emotion Regulation Scale (DERS) [28] measures participants’ emotion regulation abilities.

#### Ecological Momentary Assessment

The participants completed a series of questions numerous times a day over 28 days. The number changed across iterations. The questions aimed to (1) track mood and motivation; (2) assess goal characteristics such as reward, meaning, and importance; and (3) estimate the extent to which people are acting toward a goal, can visualize it, and feel self-efficacious.

In terms of goal pursuit, “I am acting toward a goal” was the primary outcome. Other questions measured mood, such as “How do you feel right now?” (smiley visual analog scale), anhedonia (“I’m enjoying what I am doing”), motivation (“I feel motivated), expectation (“I feel hopeful), energy (“I feel energized”), and questions related to goals, domain (“What I am doing is related to [recreation/relaxation, education, relationships, work or health”), difficulty (“What I am doing is difficult”), importance (“What I am doing is important”), reward (“What I am doing is rewarding”), meaning (“What I am doing is meaningful”), implementation (“I know how I am going to reach this goal”), and representation (“I can picture myself achieving this goal”).

#### Ecological Momentary Intervention

The study design was guided by the Risk of Bias in N-of-1 Trials (RoBiNT) scale to ensure the methodological quality of intervention studies employing single-case methodology (Multimedia Appendix 2). Details of each iteration and adaptation can be found in Multimedia Appendix 3.

### Pilot Component

Following the completion of baseline measures, the participants completed a 7-day EMA monitoring period, during which they completed 4 EMA surveys per day, occurring at semirandom intervals within an individualized 12-hour waking period (baseline). Following the completion of 7-day monitoring, the participants completed the self-guided COM-B/MCII training via the internet.

During the intervention period (the subsequent 21 days), all participants received a prompt at the start of each day, following which the goal pursuit variable alone was used to trigger prompts (falling 1 SD below their rolling average). If the individual indicated low goal pursuit, they were not asked subsequent questions relating to the goal (domain, difficulty, importance, reward, meaning, implementation, and representation), as this was considered aversive in previous iterations.

### Statistical Analyses

#### Acceptability / Feasibility

To evaluate the acceptability of study procedures, participants’ experiences were assessed in the postintervention questionnaire and analyzed descriptively.

#### EMA Analysis: Preprocessing Period

Participants with less than 48 time points were removed from the EMA analysis. In addition, participants were excluded when there was a baseline trend (ordinary least squares standardized beta coefficient >+/–0.3) on the basis that differences between phases can be difficult to interpret if improvement trends are observed in phase 1 (ie, due to natural improvement unrelated to the intervention).

#### SCED Analysis

The data obtained from the single-case experimental phase design used in this study have a hierarchical 2-level structure with observations (level 1) nested within individuals (level 2). We estimated design-comparable between-case standardized mean differences (BC-SMD) using restricted maximum likelihood methods [29]. We modeled baselines including both fixed and random effects for each level. The treatment phase was modeled with linear trends with both fixed and random effects at the level and slope. Assumptions were set around the session level error structure—an autoregressive model of order 1 (AR1) with variance differing by phase.

In addition, we estimated the differences in scores between the 2 phases using Ruscio A [30]. This metric reflects the probability that a randomly selected time point in phase 2 is larger than a randomly selected time point in phase 1 (calculated via Monte Carlo simulation: 10,000 runs) [31]. We also estimated the unstandardized difference in scores between the median values in the 2 phases.

#### Pre-Post Change

Differences between baseline and postintervention assessment measures were estimated using paired-sample *t* test.

#### Network Modeling

To explore the theoretical conceptualization of goal pursuit dynamics, we estimated a temporal network analysis. We generated 2 models, the first with mood-related variables (mood, motivation, energy, hope, and interest) and goal pursuit (as these were captured at each time point), along with a separate model with goal-specific variables (difficulty, meaning, reward, importance, implementation, and representation) and goal pursuit. These were assessed separately, as the participants only rated the goal-specific variables if their goal pursuit was >1 SD below their rolling average.

We estimated multilevel vector autoregression networks via the *mlVAR* package on R software (R Foundation for Statistical Computing) [32], using the method lmer (sequential univariate multilevel estimation) with orthogonal estimation. We then visualized the autoregression networks with the *qgraph* package [33].

Both a temporal network (how the variable is predicted by all other variables after controlling for all other temporal effects) at the previous time point and a contemporaneous network (how variables are associated at the same time point, controlling for the influence of all other variables and temporal effects) were used within the results. The model assumes stationarity; as such, all items were detrended before including them in the network.

## Results

### Overview

As the purpose of the initial 2 iterations was to inform the optimization of the EMI, only data from iteration 3 (the pilot) are reported in this section.

### Participant Characteristics

Sample characteristics are presented in Table 1. The total sample size was 73 participants (participants undertaking the EMI), with 65 (89%) completing postintervention measures. There were more female than male participants. While there was variation in ethnicity, there were no Black participants, and most of the sample were students. This was a healthy sample with low to no symptoms of depression (PHQ-9: mean 3.71, SD 3.73; range 0-15) and anxiety (GAD-7: mean 3.45, SD 3.65; range: 0-12).

**Table 1:** Sociodemographic characteristics of the study sample (N=73).

Characteristics		Value
Age (years), mean (SD)		25.05 (7.49)
Female gender, n (%)		46 (63)
**Ethnicity, n (%)**		
	Asian	27 (37)
	Chinese	15 (21)
	Mixed	1 (1)
	Other	4 (5)
	White	26 (36)
**Employment status, n (%)**		
	Employed	25 (34)
	Student	46 (63)
	Unemployed/unable to work	2 (3)

### Acceptability Assessment

**Figure 3:**
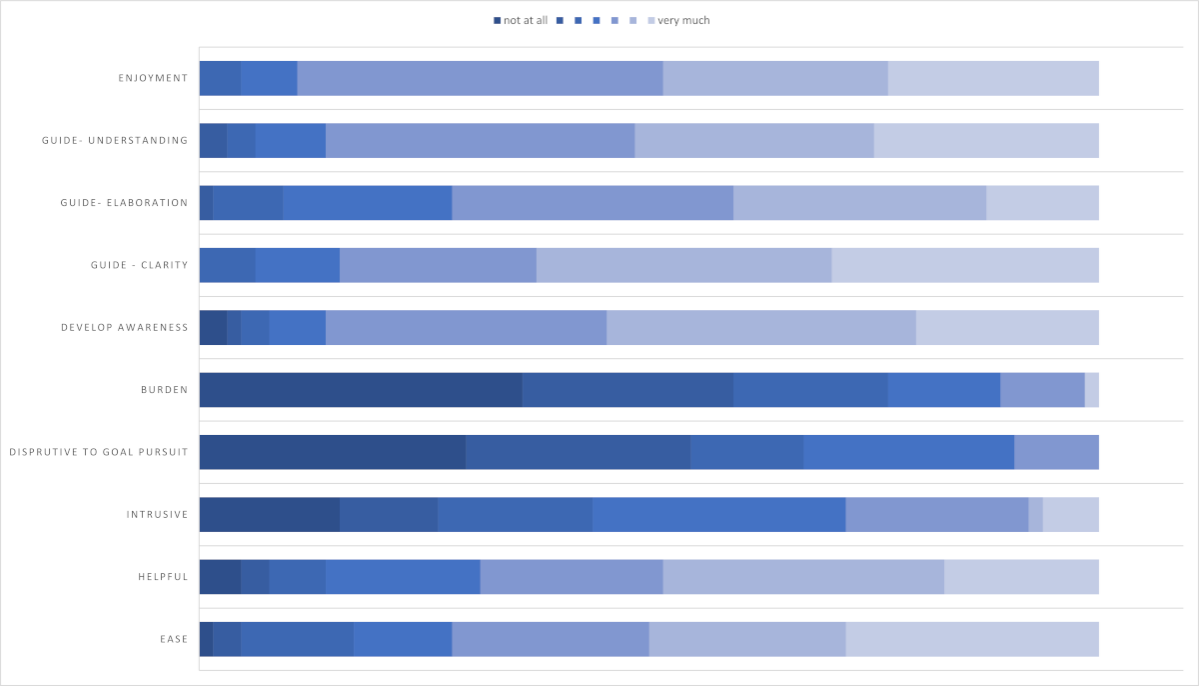
Acceptability of the intervention. This figure displays the relative frequencies of answer options.

Post intervention, the participants completed a survey on the acceptability of the intervention (Figure 3, Multimedia Appendix 4). A total of 22 (35%) respondents stated they achieved the goal “a little better than expected,” 16 (25%) reported that they achieved the goal “much better than expected,” and only 2 (3%) reported not achieving the goal or experiencing a decline in their ability to reach a desired objective. When asked to assess the general ability to effectively pursue goals, 14 (22%) participants said it became much better and 33 (52%) reported a slight improvement. Moreover, 2 (3%) participants indicated that their overall competence in accomplishing goals had deteriorated. One participant’s goal was to get a promotion, and they did not get it; it wasn’t clear why the other participant thought their competence had deteriorated).

Open feedback on user experience was largely positive. In general, participants endorsed the simplicity and interactivity of the app, which had a visualization component to help track their responses. They suggested decreasing the number of notifications during the day and making a more varied list of EMAs to avoid respondent fatigue. The participants stated the MCII strategy was helpful, and engaging with the app helped them become more aware of their own goal-related behaviors:

…Helped to keep reminding myself what I needed to accomplish and keep it in the forefront of my mind.

Participant #15

Whenever I see a reminder from the app, I seem to be persuaded to do something to change the current situation, even though I might have answered the questions with a negative emotion.

Participant #32

### Feasibility Assessment

To assess feasibility, we examined compliance with the EMA. Among all those who started the EMA (N=73), 10 (14%) were excluded from statistical analysis due to low compliance. Participants were sent 112 EMA surveys. On average, all 73 (100%) participants completed a mean of 85 (SD 33) or 76% of the EMAs across the month. While the strategy was presented at the first beep of each day, the prompt was also triggered by changes in mood on average 18 (SD 13) times per participant over a 21-day period.

### Primary Outcome: Goal Pursuit SCED Analysis

We assessed changes in goal pursuit between the baseline and intervention phases for 73 participants (Multimedia Appendix 5). Throughout the experiment, the participants’ goal pursuit domains were recreation/relaxation (1024 observations, 29%), education (882 observations, (25%), relationships (559 observations, 16%), work (581 observations, 16%), and health (508 observations, 14%). There was a small effect size (BC-SMD) 0.15, 95% CI 0.03-0.27). The intervention had an immediate significant effect, increasing the participants’ goal pursuit by 0.495 units (standard error: 0.152) (**P*<.001*) but no significant additional improvement during the intervention period, at an intervention trend of 0.002 (*P=.*002). The probability of superiority (Ruscio A) was 0.59 (95% CI 0.54-0.63). There was a large amount of heterogeneity (*I*^2^ =78.2%; H^2^=4.6), with 20 (27%) participants demonstrating CIs >0.5, 3 (4%) below 0.5 (suggesting poorer performance during the intervention phase), and the remaining 50 (69%) unclear (CIs crossing 0.5). The median difference between phases was 0.41 (SD 1.29).

### Secondary Outcomes

A total of 65 (89%) participants completed the postintervention measures. No significant pre-post changes were noted for Defeatist Performance Beliefs (DPB) (t_64_=0.36, *P*=.72*)*, Prospective Imagery Test (PIT) (t_64_=0.47, *P*=.64), DERS (t_64_=0.47, *P*=.64), MCQ (t_25_=1.36, *P=.*19), PHQ-9 (t_61_=1.65, *P*=.10*)*, and GAD-7 (t_64_= –0.24, *P=.*81). GAD-7 and PHQ-9 were affected by floor effects. On the Action Control Scale (ACS) subscales, hesitation was significant (t_64_=2.1121, **P*=.04*) but not volatility (t_61_= –0.76017, *P=*.45) or preoccupation (t_61_=1.0326, *P*=.31). There was no significant change between baseline and intervention phases for the other EMA variables: mood (BC-SMD 0.03, 95% CI –0.07 to 0.13), motivation (BC-SMD 0.03, 95 % CI –0.08 to 0.14), energy (BC-SMD –0.01, 95% CI –0.13 to 0.11), anhedonia (BC-SMD 0.01, 95% CI –0.12 to 0.10), and expectancy (BC-SMD 0.06, 95% CI –0.07 to 0.19).

### Contemporaneous Networks

**Figure 4:**
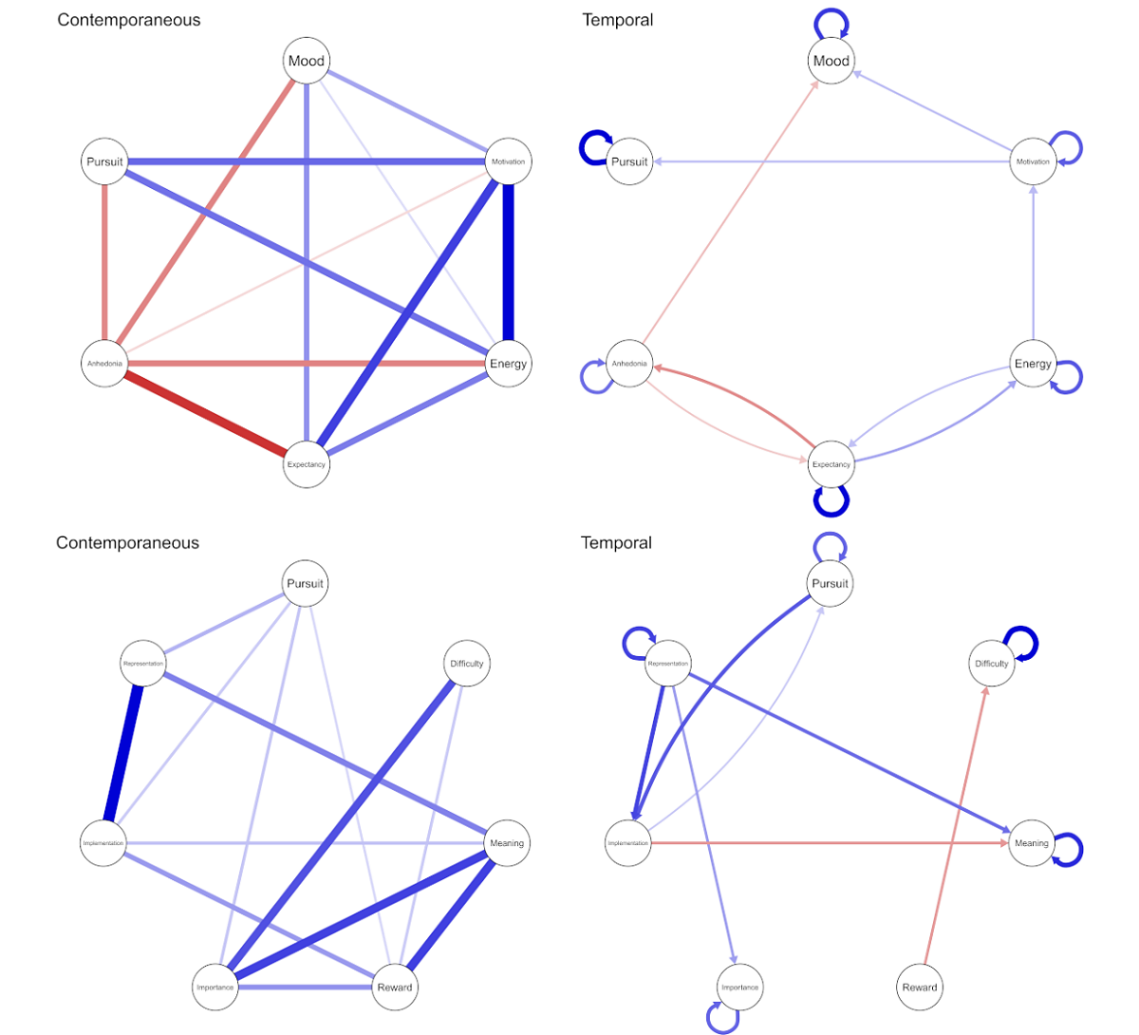
Network plots of the primary (top) and secondary (bottom) variables. Circles represent the ecological momentary assessment (EMA) items. Within the contemporaneous networks, lines represent partial correlations between symptoms at the same timepoint, after controlling for all other variables and temporal effects. In the temporal networks, directed lines indicate where a symptom predicts another symptom at the next time point after controlling for all other variables. Blue lines indicate positive relationships, and red lines indicate negative relationships. The width and saturation of a line indicate the strength of the relationship.

To elucidate dynamic processes during goal pursuit, we also estimated the network of associations between variables (Figure 4). The contemporaneous network visualized the partial correlations between variables at the same time point (controlling for the influence of all other variables and temporal effects). In the primary network, mood-related variables were associated as expected (mood, motivation, energy, and expectation) and anhedonia, motivation, and energy directly were associated with goal pursuit (explained variance for goal pursuit: *R*^2^= 0.24). Meanwhile, in the secondary network, goal-specific variables were strongly associated, with representation, implementation, importance, and reward directly associated with goal pursuit (explained variance for goal pursuit: *R*^2^= 0.07).

### Temporal networks

The temporal network demonstrated how the variables predicted each other from 1 time point to the next. Within the primary temporal network, all variables demonstrated strong autocorrelations; only motivation predicted pursuit at the next time point, with motivation predicated by energy. There was a bidirectional relationship between expectation and anhedonia, with anhedonia negatively predicting mood. Expectation and mood both influenced each other at the next time point.

Within the secondary network, there were strong autocorrelations for all variables except implementation and reward. Pursuit was predicted by implementation but had a stronger influence on implementation. Representation also predicted implementation at the next time point, as well as importance and meaning. Meanwhile, implementation negatively predicted meaning at the next time point. Reward negatively predicted difficulty, while difficulty only predicted itself.

## Discussion

### Principal Findings

This study aimed to develop, evaluate, and implement a just-in-time adaptive intervention to improve goal pursuit. Overall, the results suggest that the digital intervention was feasible and acceptable to participants. Our results show that participants endorsed high acceptability ratings relating to both the study procedures and the intervention. While there was a high level of attrition between baseline measures and those setting up the app, there was also a high level of retention and completion for those who did begin the EMI. There was a significant improvement in goal pursuit (between baseline and intervention) with most participants achieving their primary goal and reporting that they would continue using the strategy, supporting its potential effectiveness in promoting positive behavior change. There was no improvement in pre-post measures measuring processes associated with goal pursuit.

This study focuses on the dynamics of goal pursuit including the reality that people will switch between multiple goals. There is little in the intervention literature that covers dynamic within-person processes that help individuals pursue their goals over time. Similar digital intervention studies have examined the use of employing within-person dynamic data to inform prompts. Korinek et al [34] used dynamical systems modeling within their adaptive intervention to increase walking behavior in participants who were overweight to set an “ambitious but doable” goal for themselves. Fallon et al [35] used a microrandomized control design to randomize participants to an intervention option (providing goal or social feedback relating to a physical activity goal) based on each participant’s specific state. Notably, their results suggested that the effectiveness of the intervention depended on the stage of their goal pursuit (how close they were to attainment). This study contributes to the literature by using the within-person variation on goal pursuit to prompt the intervention, leading to improved goal pursuit over time.

The only change in the associated goal pursuit measures was for hesitation—the ability to initiate intended actions. This construct appeared to align with implementation intentions, looking to improve the ability to translate specific intentions into behavior. This construct has been suggested to be particularly important for goal-striving across numerous domains [36]. Given the emphasis on mental representation within the strategy, it was surprising that there was no improvement in representation. This may be due to the sensitivity of the measures, where similar measures have not been associated with task performance [37]. The measures did not capture the vividness or intensity of the imagery, which would be important phenomena underpinning scene construct [38], and were expected to be targeted. While we assessed processes related to goal pursuit, we did not assess the mechanisms of change related to all strategies. Self-monitoring, for instance, is thought to improve mental health and well-being by increasing emotional self-awareness [39,40]. Future studies should endeavor to identify measures related to the mechanisms of change.

The network analysis can inform our understanding of dynamic goal pursuit highlighting a complex cyclical process involving interdependence, influence, and self-sustaining processes. There were no changes in the associated goal pursuit measures, either in terms of pre-post measures or EMA items. Contemporaneously, motivation and reward were directly associated with goal pursuit, while anhedonia was negatively associated with it. In terms of goal-specific constructs, representation, implementation, importance, and reward were directly associated with goal pursuit. Only motivation and implementation (knowing how the goal could be achieved) predicted goal pursuit at the next time point, with goal pursuit itself being the strongest predictor of goal pursuit. This would seem to suggest that the intervention directly targets goal pursuit rather than indirectly through an associated process (eg, motivation). Indeed, within the sample, motivational levels were high. Pursuit also predicted implementation, suggesting that goal pursuit is self-sustaining and boosts confidence in knowing how to achieve the goal. This is in line with the GOAL (goal-orientated action-linking) architecture, where motivation arises from the individual’s perception that their actions can impact the likelihood of achieving a goal [1]. Self-monitoring behavior and implementing the strategy may aid goal reprioritization, where the goal pursuit itself is a driver of further goal pursuit (within or between goals), as noted by the bidirectional relationship between pursuit and implementation.

### Strengths and Limitations

The study had a few strengths and limitations. The development of the study procedure over multiple iterations facilitated adjustments including short momentary assessments, an efficient reporting process, low attrition, and high compliance. In addition to the app, the researchers provided consistent support through the intervention period, with reminder emails appearing particularly useful for improving compliance. For EMA technology to be successful in gathering accurate data and sustaining user interest over time, it is essential to engage users effectively. This is especially important when considering the translational application to mental health, where it can facilitate individuals taking a more active role in their recovery [41,42].

While there is an indication of an impact on goal pursuit, the single-case design reveals that while it might prompt change for some individuals, its effects remain unclear for the majority and entirely absent for others. In addition, the trend over the intervention did not indicate an incremental benefit; however, it may have been a sustaining pursuit, and without prompts, we may have seen a decline. Without a follow-up, it is also unclear whether the changes were maintained over time without prompts. Self-report requires self-awareness; indeed, we viewed the EMA as an active component enhancing awareness, but this could affect the measurement (either through meaning associated with items or change consequential to EMA), and this reactivity can interfere with causal claims [43].

This study aimed to personalize the just-in-time adaptive intervention. In the second iteration, we aimed to personalize the approach by identifying predictors of goal pursuit during the baseline period. However, this approach was hampered by poor compliance. In the pilot, we adopted a more conservative approach focusing solely on variation in goal pursuit; however, further studies may be able to improve the design by reinstating this approach. Finally, in relation to strategy training, the results from the initial iteration indicated that the web-based self-facilitated guide was considered optimal over video. There is evidence that the mode of learning may affect the effect size, where facilitator-led is stronger than self-facilitated [44].

Behavioral interventions that can be delivered via an app can address barriers that typically hamper engagement in intervention and may aid in study retention. Strategies for user engagement are a key aspect of EMI design. In this study, reducing participant burden was an important consideration; through iterations, we reduced the number of assessments and introduced branching of responses when not pursuing a goal. The number of assessments was still an issue for some participants, and this reduction comes at a cost to the availability and validity of data—for instance, nonrandom missing data (we had far fewer responses to model the secondary network). Passive monitoring helps reduce the burden but relies on proxy measures of goal pursuit and associated psychological processes, and it may not be as relevant for some goals as others [45].

Further research will need to consider piloting this EMI within a clinical sample before proceeding to a larger trial. The design has been optimized for easy integration into an individual’s daily life in terms of time and effort, with the intention that it could serve as an adjunct to behavioral interventions (psychological or health-related) in future research. Further considerations will need to be given to whether this should be tested as a standalone or an adjunct within an established intervention—for instance, facilitating behavioral activation for depression, or with cognitive behavioral therapy targeting the negative symptoms of psychosis. In this study, the participants were required to have enough motivation to pursue their goals. There is the question of whether this would be appropriate for those who lack motivation, as experienced in depression or psychosis. It is also unclear which aspects of the study design may need to vary, as it has been suggested that while compliance is related to fewer prompts in nonclinical samples, more frequent prompts led to higher compliance in studies with clinical samples [43]. Further research will need to carefully consider the design, intensity, and appropriateness of this EMI intervention for use within different clinical samples, while also accounting for differences in the mechanism of change between nonclinical and clinical samples.

### Conclusion

This pilot just-in-time adaptive intervention used behavior self-monitoring, COM-B, and MCII strategies to improve dynamic goal pursuit. It was delivered via an EMI procedure and shown to be feasible and acceptable among a nonclinical adult sample. Given the potential feasibility, these results provide a foundation from which future research may implement a more rigorous methodology to assess efficacy within clinical populations that experience goal pursuit deficits. There was preliminary evidence of an effect on goal pursuit. However, this should be tested in a fully powered trial before drawing conclusions. Future research should consider the utility of this approach as an additional intervention element within psychological interventions to improve goal pursuit. Sustaining goal pursuit throughout interventions is central to their effectiveness and warrants further evaluation.

## Appendix

Multimedia Appendix 1 Details of the training session.

Multimedia Appendix 2 Methodological single-case experimental design (SCED) approach based on the Risk of Bias in N-of-1 Trials (RoBiNT) scale.

Multimedia Appendix 3 Details of each iteration and adaptions.

Multimedia Appendix 4 Acceptability questions and ratings.

Multimedia Appendix 5 Ecological Momentary Assessment (EMA) descriptives.

## Abbreviations

ACS: Action Control Scale
AR1: autoregressive model of order 1
BC-SMD: between-case standardized mean difference
COM-B: Capability, Opportunity, Motivation, and Behavior framework
DPB: Defeatist Performance Beliefs
EMA: Ecological Momentary Assessment
EMI: Ecological Momentary Intervention
GAD-7: Generalized Anxiety Disorder 7
GOAL: goal-orientated action linking
MCII: mental contrasting with implementation intentions
PHQ-9: 9-item Patient Health Questionnaire
PIT: Prospective Imagery Test
RoBiNT: Risk of Bias in N-of-1 Trials
SCED: single-case experimental design

## Data Availability

The data sets and scripts generated and analyzed during this study are available in the OSF repository [46].
