# Supplementary material for: An Ecological Mobile Momentary Intervention to Support Dynamic Goal Pursuit: Feasibility and Acceptability Study
Source: JMIR Form Res. 2024 Mar 20;8:e49857. doi: 10.2196/49857 (PMC10993123; doi:10.2196/49857)
Supplement: Multimedia Appendix 1 [file formative_v8i1e49857_app1.docx]

## Details of training session

During the training period the COM-B model for behaviour change (Michie et al., 2011) and MCII strategy (Oettingen, 2012) facilitate goal setting through identifying an important and feasible goal, and assessing barriers relating to capability, opportunity, and motivation. The person then imagines a desired future outcome and mentally contrasts it with where they are at in the present, and identifies obstacles in the present that prevent goal attainment. The person then uses the implementation intentions strategy (contingency or ‘if-then’ planning) to problem solve overcoming barriers. The combination aims to create a link between the obstacle and the instrumental behaviour to overcome it, and when critical situations arise, goal-directed behaviour is immediate, automatic, and effective (Oettingen, 2012).

Participants write down their responses to every step of the exercise. First, participants identify a goal they wanted to fulfil the most in the next four weeks. These goals should require sustained effort over several weeks, but not be as long-term as running a marathon or completing a degree program. Examples of typical goals chosen by participants included completing a 10k race or studying 4 evenings a week***.*** To ensure adequate motivation, participants rate their motivation from one to ten and those with a motivation lower than seven are prompted to reconsider their goals. Second, participants are guided to vividly imagine the best outcome associated with fulfilling their goals. Third, obstacles that may impede their goal progress are considered and participants are instructed to think about their obstacles in terms of whether they have the knowledge and skills required (i.e., capacity) and whether the current environment was suitable for their goal pursuit (i.e., opportunity). Following that, they imagine one main obstacle and identify an action to overcome the obstacle to inform an if-then plan using the format “If … (obstacle) … then I will ... (action or thought to overcome the obstacle)”. It was stressed that goals change throughout the day and they could change their goals freely during the intervention, prompting them to use MCII as a daily mental strategy for general goal pursuits. Finally, the participants review the steps and reconsider any step requiring further elaboration.

References

Michie, S., van Stralen, M. M., & West, R. (2011). The behaviour change wheel: A new method for characterising and designing behaviour change interventions. *Implementation Science*, *6*(1). https://doi.org/10.1186/1748-5908-6-42

Oettingen, G. (2012). Future thought and behaviour change. *European Review of Social Psychology*, *23*(1), 1–63. https://doi.org/10.1080/10463283.2011.643698
