# Supplementary material for: An Ecological Mobile Momentary Intervention to Support Dynamic Goal Pursuit: Feasibility and Acceptability Study
Source: JMIR Form Res. 2024 Mar 20;8:e49857. doi: 10.2196/49857 (PMC10993123; doi:10.2196/49857)
Supplement: Multimedia Appendix 2 [file formative_v8i1e49857_app2.docx]

|  | | |
| --- | --- | --- |
| Item | RoBiNT Scale | SCED details, per optimisation iteration (anticipated points) |
| *Internal validity subscale* | |  |
| 1 | Design | A *replicated AB-design* with 40×A-B (total of 80 phases), providing the opportunity to observe the experimental effect 40 times. (*2 points*) |
| 2 | Randomisation | The baseline phase lasted 7 days (up to 28 timepoints) (1 *points*) |
| 3 | Sampling behaviour during all phases | The baseline phase lasted 7 days, with at least four times a day sampling, resulting in 28 *data points* (*phase A*) (assuming 100% compliance to diary). The intervention phase ran over 3 weeks, with four times a day sampling, resulting in *84 data points* (*phase B*) (assuming 100% compliance to diary). Even if the compliance rate should be lower, the amount of data points will lie >5 data points. (*2 points*) |
| 4 | Blinding of participants and HCP delivering the treatment | *Blinding* of the participant and practitioner is *not feasible*. The behavioural treatment is delivered through a web-platform independently of the researcher; however, the HCP provides technical support and encouragement. Neither the participant nor the HCP is blinded. (*0 points*) |
| 5 | Blinding (masking) of assessors | Participants complete self report assessments and receive intervention prompts and are *not blinded* to treatment phase, therefore, not independent of the therapy process. (*0 point*) |
| 6 | Inter-rater agreement | The measure of the target behaviour is a *subject ve measure* relying on *self-reports* from the digital diaries. (*0 points*) |
| 7 | Treatment adherence | The treatment is delivered through a *web-platform* following a standardised approach. Adherence to treatment (%) is calculated using *digital log-in data*. (*2 points*) |
| *External validity and interpretation subscale* | | |
| 8 | Baseline characteristics | Baseline characteristics were assessed. Furthermore, prompts in the intervention phase were informed by data collected during the baseline phase. (*2 points*) |
| 9 | Setting | The participant will engage with the online treatment in their everyday life, and therefore, it will not be possible to include details about the specific environment. (*1 point*) |
| 10 | Dependent variable (target behaviour) | **Table 2** provides an overview of all diary items, which are scores on a 10-point Likert-Scale. (*2 points*) |
| 11 | Independent variable (treatment) | A detailed description of the intervention is provided, including the *intervention content*, and frequency of intervention. (*2 points*) |
| 12 | Raw data record | *All cases* are recorded. Raw data will be presented with a data point for each diary entry. (*2 points*) |
| 13 | Data analysis | Data will be analysed and reported for each participant individually. Structured visual analysis, effect size measures and multilevel models will be applied. (2 points) |
| 14 | Replication | The study will be conducted across iterations allowing for replication of results. Across all iterations, data from n=92 participants will be available. (2 points) |
| 15 | Generalisation | Patients will be heterogeneous in their characteristics. Furthermore, retrospective self-reports will be completed by each participant *pre–post treatment* (for details, see **table 3**). (*1 point*) |

Methodological SCED approach based on the RoBiNT Scale
