# Supplementary material for: An Ecological Mobile Momentary Intervention to Support Dynamic Goal Pursuit: Feasibility and Acceptability Study
Source: JMIR Form Res. 2024 Mar 20;8:e49857. doi: 10.2196/49857 (PMC10993123; doi:10.2196/49857)
Supplement: Multimedia Appendix 3 [file formative_v8i1e49857_app3.docx]

#### Iteration 1. Push/Pull (n=10)

Following the completion of baseline measures, participants completed the self-guided COM-B/MCII training online. Over the next 28 days participants answered EMA questions, at 6 time points during the day. Questionnaire times were customized for each participant, based on their waking/sleep times, and were set at regular intervals. Participants were given 60 minutes to complete the questionnaire before it expired.

The EMI component was push/pull, participants were sent a prompt notification with a summary of the MCII strategy every morning, they could also access the guide on the home screen of the app at any time. Participants could also track their responses on the EMAs and review their total numbers of responses given, their daily average response rate, and the number of EMAs completed that day on M-path.

Following the intervention, participants completed post questionnaires and a survey on the acceptability of the procedure and intervention.

All ten participants completed the study however technical issues were experienced by two participants affecting EMA response rates (63%: 73% when removing those experiencing technical issues). Overall, the participants found the experiment acceptable.

#### Iteration 2. Personalised prompts (n=19)

Iteration 2 followed the same protocol as Iteration 1, but we added a baseline phase with the followed adaptations. Following the completion of baseline measures, participants completed a 7-day EMA monitoring period during which they complete 6 EMA surveys per day, occurring at semi-random intervals within an individualised 12-hour waking period.

The data from the 7-day monitoring was then analysed, for purposes of identifying optimal delivery of intervention prompts. Analysis of the initial 7-day EMA data include within-person summary statistics and exploratory multiple regressions, allowing identification of items predicting goal pursuit. The strongest predictor was then set as a target for triggering prompts to engage with MCII.

Following the completion of 7-day monitoring, participants completed the self-guided COM-B/MCII training online. For the following 21 days participants continue to complete EMA, and were sent EMI prompts via the smartphone app when a prompt had been triggered - if on an EMA their score on the target variable fell below a certain threshold (1SD below their rolling average).

In comparison to iteration 1, engagement was poor during this iteration (20%) with only 12/19 completing the study. Some participants reported that they found the number of EMAs intrusive and negatively affected their goal pursuit. Participants found the intervention prompts helpful and wished the experiment was more focused on the intervention.

From an experimenter perspective the identification of personalised targets was hampered by poor engagement (i.e. unreliable estimates).

#### Iteration 3 (Pilot).

Iteration 3 followed the same protocol as iteration 2, with the following adaptations: EMA surveys were reduced from 6 to 4. The initial 7 days was used for baseline measurement only (not for identifying targets). During the intervention period (the subsequent 21 days) all participants received a prompt at the start of each day, as previous respondents requested more focus on the intervention component, following which, the goal pursuit variable alone was used to trigger prompts (falling 1SD below their rolling average). If the individual indicated low goal pursuit they were not asked subsequent questions relating to the goal (domain, difficulty, importance, reward, meaning, implementation, representation), as this was considered aversive in previous iterations. The poor engagement also highlighted the need for more collaborative relationship between researcher and participant which was addressed by researchers providing feedback on compliance and helping with any issues (up to two times where dropping below 80% compliance) and built in app rewards (badges) for active participation
