# Supplementary material for: An Ecological Mobile Momentary Intervention to Support Dynamic Goal Pursuit: Feasibility and Acceptability Study
Source: JMIR Form Res. 2024 Mar 20;8:e49857. doi: 10.2196/49857 (PMC10993123; doi:10.2196/49857)
Supplement: Multimedia Appendix 4 [file formative_v8i1e49857_app4.docx]

Table 2. Acceptability questions and ratings.

| Question | M(SD) [range] |
| --- | --- |
| Did you achieve the goal? | 4.5(1.6) [1-7] |
| How would you rate the improvement in your ability to purse goals? | 4.9(1.2) [1-7] |
| Overall, did you enjoy the experiment? | 5.6(1.1) [3-7] |
| Did the online guide explain the strategy clearly enough? | 5.7(1.2) [3-7] |
| Did the strategy make you more aware of your own behaviour? | 5.5(1.3) [1-7] |
| Was the online guide sufficient for you to fully understand the strategy? | 5.5(1.2) [2-7] |
| Was the online guide useful to help you elaborate you goals and obstacles? | 5.1(1.2) [2-7] |
| Did answering the questions take you too much time every day? | 2.4(1.5) [1-7] |
| Did receiving/answering the questions disrupt your goal pursuit? | 2.6(1.4) [1-5] |
| How intrusive did you find the messages? | 3.6(1.6) [1-7] |
| Was it easy to respond to the questions through the day? | 5.3(1.6) [1-7] |
| Were the strategy reminders useful to help you pursue your goals? | 5.1(1.6) [1-7] |
| Will you continue to use this strategy? | Yes: 48 (76%) |
| Would you recommend this strategy to a friend? | Yes: 54 (86%) |
