# Supplementary material for: An Ecological Mobile Momentary Intervention to Support Dynamic Goal Pursuit: Feasibility and Acceptability Study
Source: JMIR Form Res. 2024 Mar 20;8:e49857. doi: 10.2196/49857 (PMC10993123; doi:10.2196/49857)
Supplement: Multimedia Appendix 5 [file formative_v8i1e49857_app5.docx]

EMA descriptives. SD= standard deviation; MAD = median absolute deviation

|  | Baseline |  |  | Intervention | |
| --- | --- | --- | --- | --- | --- |
|  | Mean (SD) | Median (MAD) | | Mean (SD) | Median (MAD) |
| Mood | 6.73(0.93) | 6.71(0.77) | | 6.73(0.94) | 6.82(0.62) |
| Motivation | 5.81(1.49) | 5.82(1.64) | | 5.84(1.73) | 6.07(1.38) |
| Energy | 5.82(1.35) | 5.78(0.97) | | 5.79(1.67) | 5.96(1.77) |
| Energy | 3.63(1.1) | 3.76(0.91) | | 3.63(1.2) | 3.50(1.45) |
| Pursuit | 6.13(1.41) | 5.93(1.27) | | 6.48(1.33) | 6.55(1.35) |
| Expectancy | 3.75(1.22) | 4.04(1.16) | | 3.89(1.6) | 3.65(1.36) |
